# Supplementary figures and images for: The hyaluronan receptor CD44 drives COVID-19 severity through its regulation of neutrophil migration
Source: PLoS Pathog. 2026 May 20;22(5):e1013619. doi: 10.1371/journal.ppat.1013619 (PMC13221140; doi:10.1371/journal.ppat.1013619)

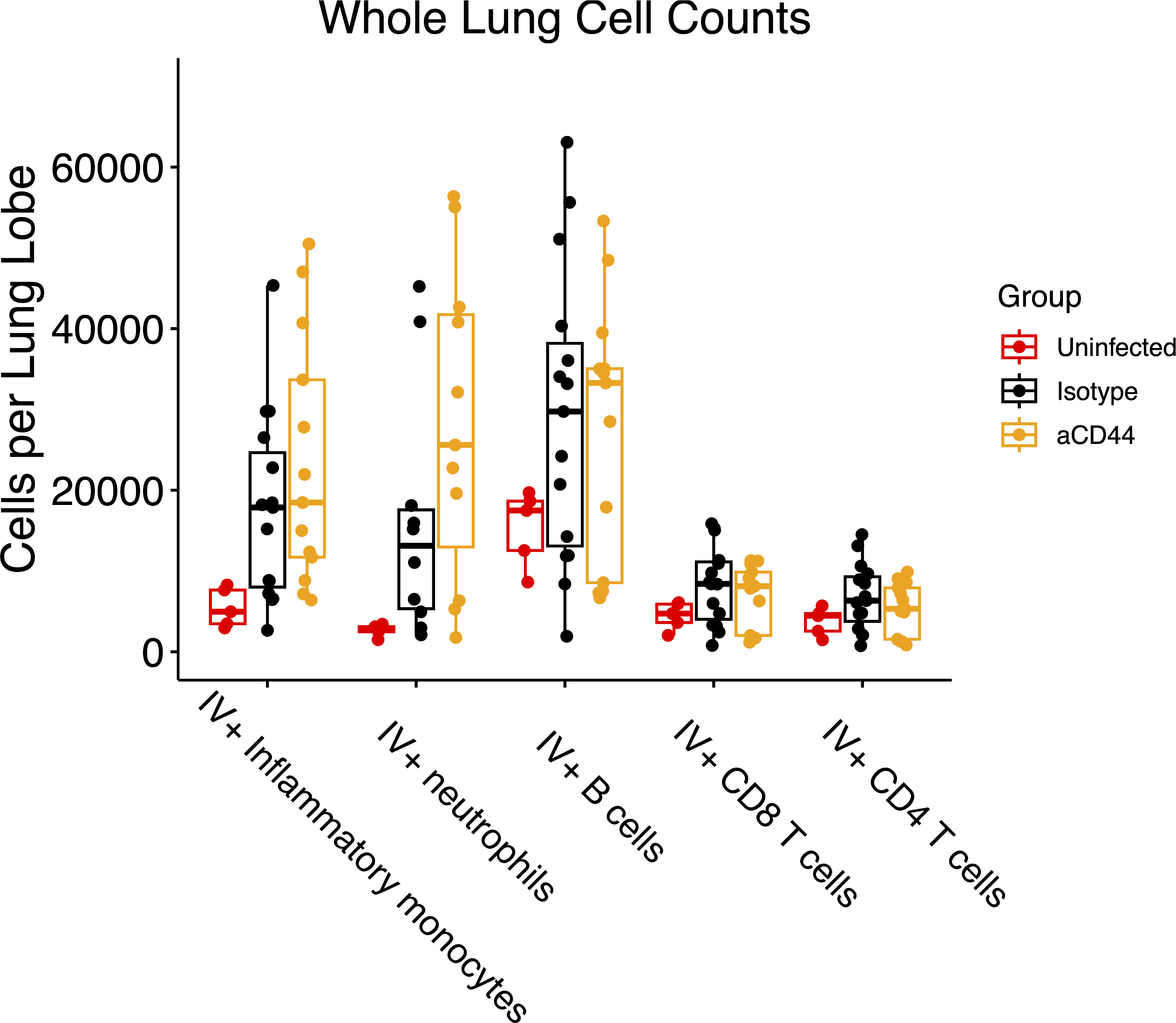

Supplement: S2 Fig — Changes in circulating lymphocyte numbers in whole lung homogenates taken from MA10-infected mice on day 4 post infection. Retro-orbital anti-CD45 was administered 5 minutes before euthanization. Cells that stain positive for this IV-anti-CD45 were marked as IV+. Neutrophils (CD45+ CD11b+ CD11c- Ly6Chi Ly6G+), Inflammatory monocytes (CD45+ CD11b+ CD11c- Ly6Chi Ly6G-), B cells (CD45+ CD19+ SSClo), CD8 T cells (CD45+ CD3+ CD8+ SSClo), and CD4 T cells (CD45+ CD3+ CD4+ SSClo) are shown. No significant differences in P values calculated via one way ANOVA followed by Tukey’s HSD test. (TIF) [file ppat.1013619.s002.tif]

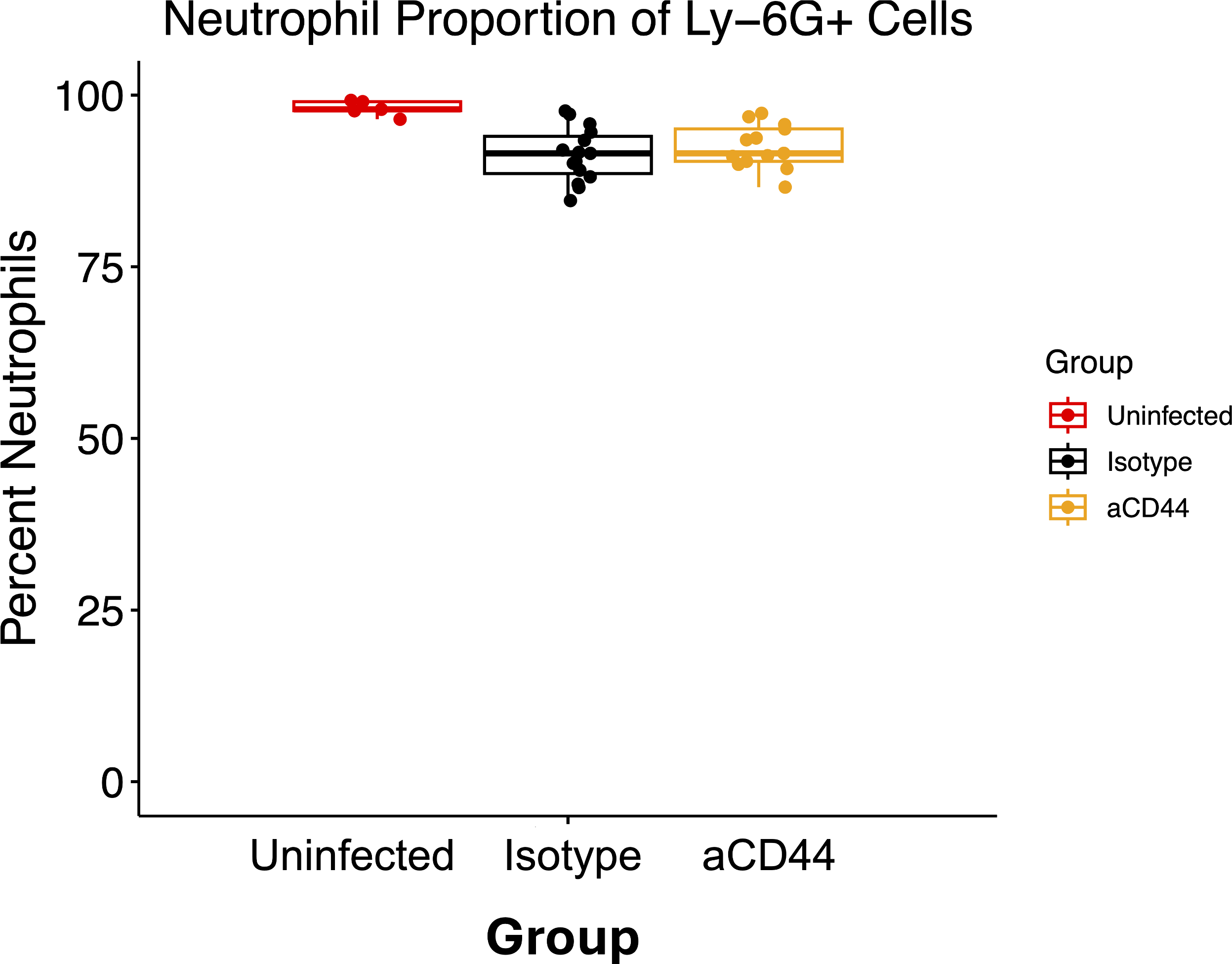

Supplement: S5 Fig — Neutrophils (CD45+ CD11b+ CD11c- Ly6Chi Ly6G+) as percentage of all live Ly-6G+ cells in whole lung homogenates taken from MA10-infected mice on day 4. (TIF) [file ppat.1013619.s005.tif]
