# Supplementary material for: The hyaluronan receptor CD44 drives COVID-19 severity through its regulation of neutrophil migration
Source: PLoS Pathog. 2026 May 20;22(5):e1013619. doi: 10.1371/journal.ppat.1013619 (PMC13221140; doi:10.1371/journal.ppat.1013619)
Supplement: S1 Table — (DOCX) [file ppat.1013619.s006.docx]

| **Target** | **Fluorophore** | **Host Species** | **Target Species** | **Source** | **Catalog Number** | **Dilution** |
| --- | --- | --- | --- | --- | --- | --- |
| HA | Biotin | Human | Mouse | Echelon Biosciences | G-HA02 | 1:100 |
| SARS-CoV-2 N protein |  | Rabbit | SARS-CoV-2 | GeneTex | GTX135357 | 1:800 |
| CD45 |  | Goat | Mouse | R&D Systems | AF114-SP | 1:300 |
| Rabbit-anti-mouse Ly-6G (IF) |  | Rabbit | Mouse | Thermo Fisher Scientific | MA5-51254 | 1:200 |
| Streptavidin | Texas Red |  | Biotin | Vector Laboratories | SA-5006-1 | 1:1000 |
| Donkey-anti-Rabbit Secondary | FITC | Donkey | Rabbit | Jackson ImmunoResearch | 127-095-099 | 1:1000 |
| Donkey-anti-Goat Secondary | Cy5 | Donkey | Goat | Jackson ImmunoResearch | 111-175-144 | 1:1000 |
