# Supplementary material for: The hyaluronan receptor CD44 drives COVID-19 severity through its regulation of neutrophil migration
Source: PLoS Pathog. 2026 May 20;22(5):e1013619. doi: 10.1371/journal.ppat.1013619 (PMC13221140; doi:10.1371/journal.ppat.1013619)
Supplement: S2 Table — (DOCX) [file ppat.1013619.s007.docx]

| **Marker** | **Fluorophore** | **Sources** | **Catalog Number** | **Dilution** |
| --- | --- | --- | --- | --- |
| General Immunophenotyping | | | | |
| Live Dead | Zombie NIR | BioLegend | 423106 | 1:100 |
| CD4 | PE-Cy7 | BioLegend | 100422 | 1:100 |
| CD3 | PE-Fire 700 | BioLegend | 100272 | 1:50 |
| CD8a | Alexa Fluor 700 | BioLegend | 100730 | 1:100 |
| CD161 | Brilliant Violet 421 | BioLegend | 108732 | 1:100 |
| CD45 | PerCP-Cy5.5 | BioLegend | 103132 | 1:200 |
| CD19 | Brilliant Violet 785 | BioLegend | 115543 | 1:100 |
| CD11c | PE-Cy5 | BioLegend | 117316 | 1:200 |
| Ly-6C | FITC | BioLegend | 128006 | 1:100 |
| Ly-6G | Brilliant Violet 650 | BioLegend | 127641 | 1:100 |
| Siglec-F | PE | BioLegend | 155506 | 1:100 |
| MerTK | PE-Dazzle 594 | BioLegend | 151524 | 1:100 |
| CD11b | Brilliant Violet 480 | BD Biosciences/Fisher | 566117 | 1:100 |
| CD64 | Brilliant Violet 711 | BioLegend | 139311 | 1:50 |
| Neutrophil Specific for Supplementary Figure 4 | | | | |
| CD62L | Alexa Fluor 700 | BioLegend | 104426 | 1:100 |
| CXCR4 | PE-Dazzle 594 | BioLegend | 146513 | 1:100 |
| CD47 | RealBlue 744 | BD Biosciences/Fisher | 757182 | 1:100 |
| CXCR2 | Brilliant Violet 750 | BD Biosciences/Fisher | 747816 | 1:100 |
| MHCII | Pacific Blue | BioLegend | 107619 | 1:50 |
| ICAM-1 | PE-Cy7 | BioLegend | 116121 | 1:100 |
| CXCR1 | PE | BD Biosciences/Fisher | 566383 | 1:100 |
